# The Impact of NUTRItional Status at First Medical Oncology Visit on Clinical Outcomes: The NUTRIONCO Study

**DOI:** 10.3390/cancers15123206

**Published:** 2023-06-15

**Authors:** Maurizio Muscaritoli, Alessandra Modena, Matteo Valerio, Paolo Marchetti, Roberto Magarotto, Silvia Quadrini, Filomena Narducci, Giuseppe Tonini, Teresa Grassani, Luigi Cavanna, Camilla Di Nunzio, Chiara Citterio, Marcella Occelli, Antonia Strippoli, Bruno Chiurazzi, Antonio Frassoldati, Giuseppe Altavilla, Antonio Lucenti, Fabrizio Nicolis, Stefania Gori

**Affiliations:** 1 Department of Clinical Medicine, Sapienza University of Rome, 00185 Rome, Italy; 2 Medical Oncology Unit, IRCCS Sacro Cuore Don Calabria, 37024 Negrar di Valpolicella, Italy; alessandra.modena@sacrocuore.it (A.M.); matteo.valerio@sacrocuore.it (M.V.); roberto.magarotto@sacrocuore.it (R.M.); stefania.gori@sacrocuore.it (S.G.); 3 IDI-IRCCS, 00167 Rome, Italy; paolo.marchetti@uniroma1.it; 4 Medical Oncology Unit, S.S. Trinità Hospital, 03039 Sora, Italy; silviaquadrini@icloud.com (S.Q.); menanarducci@yahoo.it (F.N.); 5 Medical Oncology Unit, Fondazione Policlinico Universitario Campus Bio-Medico, 00128 Rome, Italy; g.tonini@unicampus.it (G.T.); t.grassani@policlinicocampus.it (T.G.); 6 Department of Medicine and Surgery, Università Campus Bio-Medico di Roma, 00128 Rome, Italy; 7 Department of Oncology-Hematology, Guglielmo da Saliceto Hospital, 29121 Piacenza, Italy; luigicavanna53@gmail.com (L.C.); c.dinunzio@ausl.pc.it (C.D.N.); c.citterio@ausl.pc.it (C.C.); 8 Department of Oncology, Santa Croce e Carle General Hospital, 12100 Cuneo, Italy; marcellaoccelli@gmail.com; 9 Medical Oncology Unit, Fondazione Policlinico Universitario A. Gemelli IRCCS, 00168 Rome, Italy; antonia.strippoli@policlinicogemelli.it; 10 Oncology Unit, Antonio Cardarelli Hospital, 80131 Naples, Italy; bruno.chiurazzi@aocardarelli.it; 11 Clinical Oncology Unit, S. Anna University Hospital, 44124 Cona-Ferrara, Italy; a.frassoldati@ospfe.it; 12 Medical Oncology Unit, Department of Human Pathology of Adult and Evolutive Age “G. Barresi”, University of Messina, 98125 Messina, Italy; galtavilla@unime.it; 13 Medical Oncology Unit, Maria Paternò-Arezzo Hospital, 97100 Ragusa, Italy; antonio.lucenti@asp.rg.it; 14 Medical Direction, IRCCS Sacro Cuore Don Calabria, 37024 Negrar di Valpolicella, Italy; fabrizio.nicolis@sacrocuore.it; 15 AIOM Foundation, 20133 Milano, Italy

**Keywords:** anorexia, malnutrition, cancer, cachexia, outcomes, awareness, survival, early management

## Abstract

**Simple Summary:**

The term “malnutrition” indicates imbalances in energy and/or nutrient intake. Cancer-related malnutrition/cachexia results from a combination of anorexia and metabolism alterations caused by the tumor itself or by its treatment, and is characterized by inflammation, increased protein breakdown, and severe loss of skeletal muscle mass. Cancer cachexia negatively affects patients’ anticancer treatment, outcomes, quality of life, and survival. However, malnutrition and risk of malnutrition are still neglected in cancer patients. The PreMiO study revealed that 51% of patients already presented with nutritional deficiencies at their first medical oncology visit. Here, we report the data obtained in the subsequent, retrospective NUTRIONCO study, revealing a significant association between the baseline clinical and nutritional characteristics collected in the PreMiO study and the outcomes at follow-up in the same group of patients. These results highlight the importance of proactive, early management of malnutrition in cancer patients.

**Abstract:**

Malnutrition affects up to 75% of cancer patients and results from a combination of anorexia and metabolic dysregulation. Metabolic and nutritional abnormalities in cancer patients can lead to cachexia, a multifactorial syndrome characterized by involuntary loss of skeletal muscle mass, systemic inflammation and increased protein catabolism. Cancer cachexia negatively affects patients’ outcomes, response to anticancer treatments, quality of life, and survival. However, risk of malnutrition, and cachexia are still under-recognized in cancer patients. The Prevalence of Malnutrition in Oncology (PreMiO) study revealed that 51% of patients already had nutritional deficiencies at their first medical oncology visit. Here, we report the results of the subsequent retrospective, observational NUTRItional status at first medical oncology visit ON Clinical Outcomes (NUTRIONCO) study, aimed at assessing the impact of baseline nutritional and non-nutritional variables collected in the PreMiO study on the clinical outcomes of the same patients followed up from August 2019 to October 2021. We have highlighted a statistically significant association between baseline variables and patient death, rehospitalization, treatment toxicity, and disease progression at follow-up. We found a higher overall survival probability in the well-nourished general study population vs. malnourished patients (*p* < 0.001). Of major interest is the fact that patient stratification revealed that malnutrition decreased survival probability in non-metastatic patients but not in metastatic patients (*p* < 0.001). Multivariate analysis confirmed that baseline malnutrition (*p* = 0.004) and VAS score for appetite loss (*p* = 0.0104), in addition to albumin < 35 g/L (*p* < 0.0001) and neutrophil/lymphocyte ratio > 3 (*p* = 0.0007), were independently associated with the death of non-metastatic patients at follow-up. These findings highlight the importance of proactive, early management of malnutrition and cachexia in cancer patients, and in particular, in non-metastatic patients, from the perspective of a substantial improvement of their clinical outcomes.

## 1. Introduction

According to the definition issued by the World Health Organization (WHO), the term “malnutrition” refers to “deficiencies, excesses or imbalances in a person’s intake of energy and/or nutrients” [1]. The term malnutrition concerns both overnutrition, leading to overweight and obesity, which are recognized risk factors for a series of pathological conditions, including the onset and progression of hormone-dependent breast and endometrial cancer [2], as well as undernutrition and nutritional risk. However, the term “malnutrition” is often used synonymously with “undernutrition”, as in this research paper. Malnutrition is common, can affect all age groups, and is both a cause and a consequence of disease; nonetheless, it is often overlooked by clinicians [3]. Cancer-related malnutrition can affect up to 75% of patients, depending on patient age, tumor type and stage, and anticancer treatment [4], and differs from starvation-related malnutrition, as it results from a combination of anorexia and metabolic dysregulation, caused by the tumor itself or by its treatment [5]. Anorexia can be described as a significant decrease in food intake resulting from altered appetite signals caused by the tumor itself or by anticancer therapies, as well as from physical issues (e.g., mouth ulcers, pain) [6]. Reduced food intake is a predictor of a high likelihood of severe weight loss in cancer patients, as recently demonstrated by an international multicenter study [7], also revealing an association between reduced food intake and cancer-related overall survival (OS).

Metabolic and nutritional abnormalities in cancer patients are the drivers of cachexia, a multifactorial syndrome considered a comorbidity of cancer and characterized by an involuntary, severe loss of skeletal muscle mass, with or without loss of adipose tissue, systemic inflammation [8], and negative protein-energy balance [9,10,11]. Cachexia includes a spectrum of manifestations, ranging from pre-cachexia, characterized by clinical symptoms and changes in metabolic biomarkers, to refractory cachexia, characterized by dramatic, unmanageable weight loss [11,12]. The progression of anorexia and cancer cachexia can lead to sarcopenia, occurring in 20–70% of cancer patients depending on the tumor type [13]. Sarcopenia is characterized by the depletion of lean muscle mass, with impaired physical function, asthenia and fatigue, reduced tolerance to treatments, impaired QoL, and reduced survival. Sarcopenia also increases the risk of toxicity of several anticancer drugs [13,14].

The agreed criterion for a diagnosis of cachexia in cancer is a weight loss >5%, or a body mass index (BMI) < 20 kg/m^2^ with a weight loss >2% body weight, or sarcopenia with a weight loss >2% body weight [15]. Cancer cachexia can affect 50–80% of cancer patients, with negative impacts on quality of life (QoL) and prognosis, and is responsible for the death of at least 20% of them [14].

Malnutrition in cancer patients leads to prognostic, but also socioeconomic consequences, and this is particularly evident in patients affected by solid neoplasms [16]. Of major interest is the revelation of the multicenter, prospective Prevalence of Malnutrition in Oncology (PreMiO) study that 51% of patients already presented with nutritional impairment at their first medical oncology visit, and that 43% were at risk for malnutrition [6]. The PreMiO study also revealed a positive correlation between stage of cancer at first medical oncology visit and severity of malnutrition. Consistently, a recent investigation revealed a high prevalence of anorexia (57%) and cachexia (68%) in patients with gastrointestinal or lung cancer, from the point of diagnosis [17].

To date, the impact of baseline nutritional deficiencies on specific oncological outcomes, including disease progression, treatment-related emerging toxicities, patient rehospitalization, and death, has not been well established. We performed the retrospective, multicenter, observational NUTRItional status at first medical oncology visit ON Clinical Outcomes (NUTRIONCO) study, which included a subset of the 1952 adult cancer patients originally enrolled in the PreMiO study [6], to assess the impact of a set of baseline clinical variables, including systemic inflammation, nutritional status and anorexia, assessed at the first medical oncology visit, on the main clinical oncological outcomes at follow-up.

## 2. Materials and Methods

### 2.1. Study Population

The retrospective, observational NUTRIONCO study included a subpopulation (*n* = 571 cancer patients) of the prospective, observational PREMiO study (*n* = 1952 cancer patients) for whom follow-up data were collected from August 2019 to October 2021 and made available in electronic case report forms (eCRF) at ten medical oncology sites in Italy.

Inclusion criteria of the NUTRIONCO study were the same applied for the previous PreMiO study: patients at first medical oncology visit; diagnosis of solid tumor; age > 18 years; no previous anticancer therapies; life expectancy >3 months and informed consent to study participation. Cancer type and stage of disease were determined by the oncologist. Exclusion criteria were inability to feed orally or intestinal obstruction; decompensated metabolic disorders; severe liver failure (total bilirubin > 1.5 mg/dL) and aspartate aminotransferase to alanine aminotransferase ratio > 2-times the upper limit normal (ULN) or, in case of metastatic liver cancer, >5 times ULN; severe kidney failure with creatinine >2.0 mg/dL (177 μmol/L) or with creatinine clearance (ClCr) < 50 mL/min; primary brain tumor or metastatic brain tumors; active infection; acute decompensated heart failure; severe psychiatric disorders; Mini-Mental State Examination (MMSE) score < 25/30 in patients aged >70 years [6].

### 2.2. Study Design and Objective

NUTRIONCO was a multicenter, retrospective observational study based on a post hoc analysis aimed at analyzing the potential association between baseline characteristics assessed at the first oncological visit in patients enrolled in the PreMiO study described above [6] and the main clinical outcomes at follow-up. The data of interest were collected from the patients’ eCRFs completed during the visits done every 12 months after the first medical oncology visit, until the date of data cut-off of the NUTRIONCO study (14 October 2021) or a patient’s death.

The baseline variables collected in the PREMiO study database that were considered by the NUTRIONCO study investigators as potential influencing factors of the clinical outcomes assessed in the NUTRIONCO study were serum levels of hemoglobin and albumin, the neutrophil/lymphocyte ratio (NLR), the Functional Assessment of Anorexia-Cachexia Therapy (FAACT) total score, the visual analog scale (VAS) for appetite loss score, the nutritional status, and the metastatic status.

The clinical outcomes collected at the date of data cut-off of the NUTRIONCO study that were considered for the association analysis with the baseline clinical and nutritional variables listed above were patient rehospitalization, onset of treatment-related toxicity, patient death, and disease progression.

### 2.3. Database Set-Up and Data Collection

Patients’ information was recorded on a data collection sheet at the time of enrollment in the PreMiO study and then uploaded to a dedicated website platform. Anonymity was maintained by assigning each patient a study identification number. Patients were stratified by cancer type/site and disease stage, as well as by age, sex, and general health condition.

### 2.4. Malnutrition- and Anorexia-Related Scores

Malnutrition and risk of malnutrition in patients enrolled both in the PreMiO study and, subsequently, in the NUTRIONCO study, were assessed through the Mini Nutritional Assessment (MNA^®^) tool. MNA^®^ is a validated, rapid nutritional screening and assessment test commonly used in cancer patients and based on a questionnaire of simple administration, not requiring a trained nutritionist. The full version of MNA^®^ includes 18 items in four categories: general, anthropometric, dietary, and subjective assessment [18]. Malnourished subjects have MNA^®^ scores < 17, those at risk of malnutrition have scores ranging from 17 to 23.5, and well-nourished patients obtain scores >23.5.

Anorexia (intended as appetite loss) was evaluated through two methods. In the first, any appetite loss was determined through a modified version of anorexia–cachexia subscale (AC/S-12) of the FAACT questionnaire. The FAACT score quantifies the patient perception of symptoms and signs that correlate with anorexia and consists of 12 questions related to appetite and food intake, allowing a qualitative and quantitative diagnosis of anorexia. Answers to each question are on a 5-point Likert scale (i.e., not at all, a little bit, somewhat, quite a bit, very much), with a corresponding score ranging from 0 to 4 [19]. In the second method, appetite loss was quantified on a VAS [20]. The VAS score represented patients’ self-perception of appetite, with patients self-reporting the oral food intake on a VAS scale ranging from 0 (no food intake) to 100 (normal food intake).

The FAACT and VAS scores for the 571 cancer patients included in the NUTRIONCO study and collected during the PreMiO study at the first medical oncology visit, were categorized considering two cut-offs: a FAACT score ≤ 30 for anorexia and a VAS score ≤ 70 for appetite loss representative of anorexia.

All evaluations of nutritional status described above were performed by an oncologist or a senior resident in oncology trained to use the nutrition assessment tools.

### 2.5. Pre-Cachexia and Cachexia Determination at Baseline

Pre-cachexia is a disease-associated condition characterized by unintentional weight loss <5% during prior 6 months, along with chronic systemic inflammation and anorexia-related symptoms. Baseline systemic inflammation was identified as albumin <35 g/L and NLR > 3 [21,22].

Anorexia-related symptoms were determined using the VAS and FAACT tools defined above. Cachexia was identified based on criteria defined by Fearon et al. [15].

### 2.6. Statistical Analysis

We used descriptive statistics to summarize the baseline characteristics of the PreMiO study patients that entered in the NUTRIONCO study. Numeric variables were presented as mean and standard deviation. Categorical variables were presented as frequency values calculated on the non-missing data collected in the database. Baseline clinical and nutritional data were categorized into binary variables (above/below the cut-off value, malnourished and/or at risk of malnutrition/well-nourished, metastatic/non-metastatic status). The inferential analyses aimed at the identification of the significant associations between categorical variables at baseline and clinical outcomes at the date of NUTRIONCO database closure, were conducted by single factor through the chi-square test of independence. *p*-values < 0.05 were considered statistically significant. Overall survival estimates and survival probabilities were obtained by applying Kaplan–Meier curves, and the comparison between survival curves was conducted using the log-rank test. The independent association between the baseline variables of the study listed above and the clinical outcome, “patient’s death”, was investigated using the multivariate Cox proportional hazards regression model, with the results presented as hazard ratios (HR) with 95% confidence intervals (CI). *p*-values < 0.05 were considered statistically significant. The SAS^®^ analytics software, version 9.4, was used for statistical analyses.

## 3. Results

### 3.1. Patients’ Demographics and Baseline Characteristics: A Comparison between the PreMiO and the NUTRIONCO Study Populations

Between 19 August 2019 and 14 October 2021, 571 out of the 1952 patients originally included in the PreMiO study [6] were followed up at 10 medical oncology sites in Italy in the setting of the retrospective, multicenter, observational NUTRIONCO study.

The baseline demographics, nutritional and clinical features and laboratory data of cancer patients included in the NUTRIONCO study are summarized in Table 1 and Table 2. The mean age of the study sample was 63.7 ± 12.7 years. The study included 262 (45.9%) males and 309 (54.1%) females. The mean BMI was 24.8 ± 4.6 kg/m^2^.

A direct comparison of baseline demographics, nutritional and clinical features and laboratory data between patients included in the PreMiO and NUTRIONCO studies highlighted substantially homogeneous characteristics for the two populations (Table 1 and Table 2). In particular, the mean serum concentration of albumin was 36.4 g/L and 36.3 g/L in the NUTRIONCO and in the PreMiO study, respectively (Table 2), above the cut-off value for healthy subjects (≥35 g/L), while the mean NLR value was 3.7 and 3.9 in the NUTRIONCO and in the PreMiO study, respectively (Table 2), slightly above the normal reference range for healthy subjects (1–3).

Mean hemoglobin concentration in male patients was 12.7 g/dL and 12.4 g/dL in the NUTRIONCO and in the PreMiO study, respectively, while mean hemoglobin concentration in female patients was 12.5 g/dL and 12.2 g/dL in the NUTRIONCO and in the PreMiO study (Table 2), i.e., proximal or slightly below the cut-off value for healthy subjects (≥13 g/dL for males and ≥12 g/dL for females)

The baseline mean BMI value was 24.8 kg/m^2^ for both the cancer patients included in the NUTRIONCO and in the PreMiO studies (Table 1), a value > 20 kg/m^2^, considered as a cut-off for the definition of cachexia, as described above [15].

**Figure 1:**
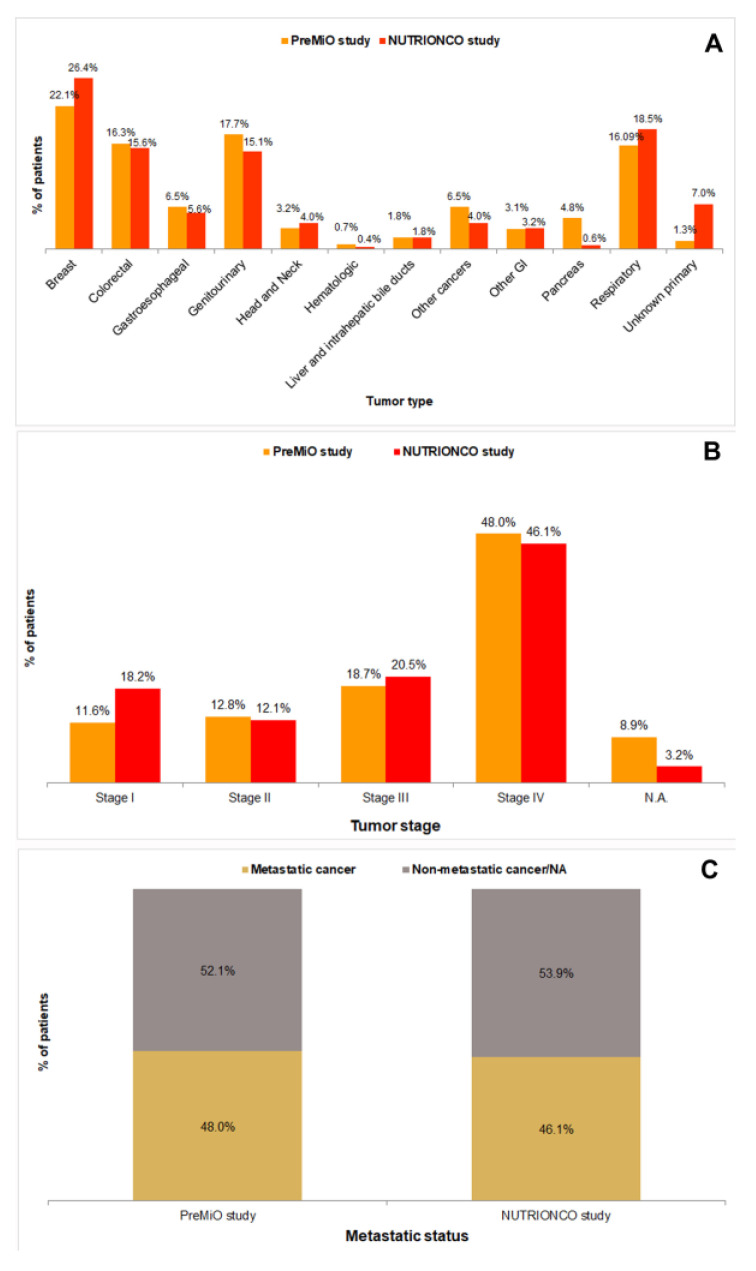
Distribution of tumor types (**A**), tumor stages (**B**), and metastatic status (**C**) in patients included in the PreMiO (*n* = 1952) and NUTRIONCO (*n* = 571) studies.

Patients populations included in the PreMiO and NUTRIONCO studies were substantially homogenous with reference to distribution of tumor types (Figure 1A), with the exception of pancreatic cancer (4.8% in PREMiO study vs. 0.6% in NUTRIONCO study) and unknown primary tumors (1.3% in PREMiO study vs. 7.0% in NUTRIONCO study). Tumor staging (I to IV) was substantially overlapping for patients’ populations included in the PreMiO and NUTRIONCO studies (Figure 1B). Similar data have been observed also for the metastatic status (Figure 1C).

For what concerns the anorexia/malnutrition-related parameters considered in the study, a baseline mean FAACT total score of 31 and 29.9 was measured for participants in the NUTRIONCO and the PreMiO studies, respectively, i.e., around the cut-off score of 30, indicative of anorexia (Table 2). In addition, a baseline mean VAS score for appetite loss of 69.8 and 67.0 was measured for participants to the NUTRIONCO and the PreMiO studies, respectively, i.e., below the cut-off score of 70 indicative of anorexia (Table 2). A FAACT total score ≤30 was more common among patients affected by respiratory tumors (19.2%), breast cancer (17.2%), and colorectal cancer (15.7%) (Table 3). The evaluation of appetite loss obtained through the VAS produced results overlapping with data from the FAACT tool: a score ≤70 was more common among patients affected by breast cancer (20.1%), respiratory tumors (18.0%) and colorectal cancer (16.2%) (Table 3).

**Figure 2:**
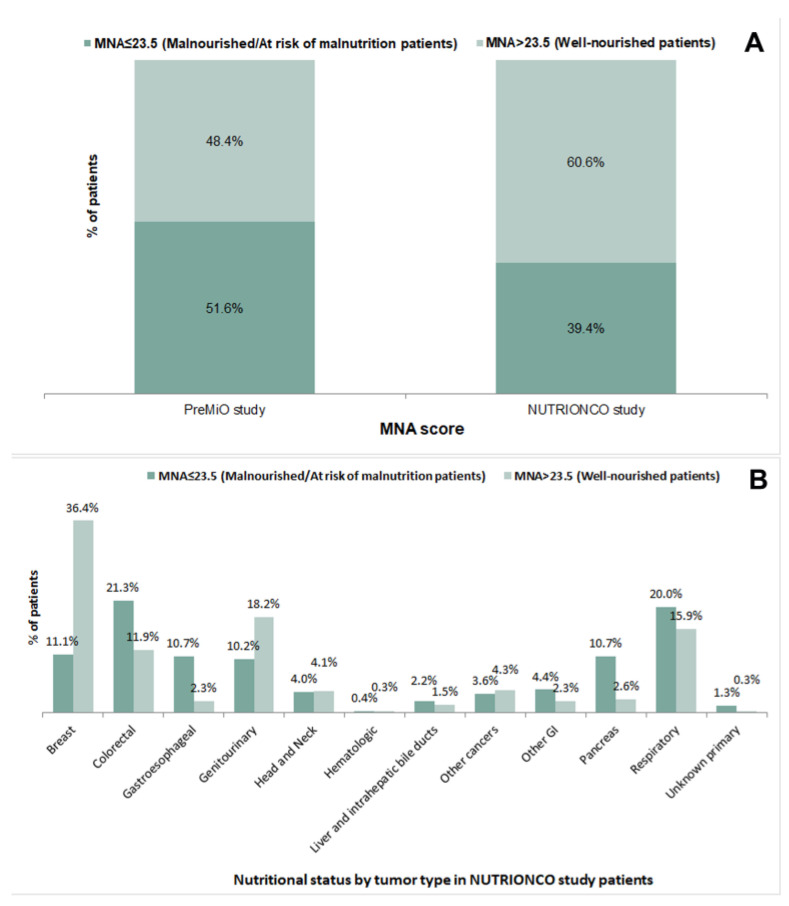
MNA^®^ score in patients included in the PreMiO (*n* = 1952) and NUTRIONCO (*n* = 571) studies (**A**) and nutritional status by tumor type as per MNA^®^ score in NUTRIONCO study patients (**B**).

About half of patients (51.6%) included in the PreMiO study obtained a baseline MNA^®^ score < 23.5 (indicating malnutrition or risk of malnutrition), vs. 39.4% of patients included in the NUTRIONCO study (Figure 2A). Malnutrition or risk of malnutrition were more common among patients of NUTRIONCO study affected by colorectal (21.3%), gastroesophageal (10.7%), pancreatic (10.7%), and respiratory cancer (20.0%) (Figure 2B).

### 3.2. Baseline Characteristics and Clinical Outcomes: An Association Analysis in the NUTRIONCO Study Population

The comparison reported above between the baseline demographics and clinical and nutritional variables of the population included in the PreMiO study and of its subpopulation included in the NUTRIONCO study revealed a substantial homogeneity of the two groups of cancer patients. On this basis, we performed an association analysis between the baseline clinical (i.e., hemoglobin, albumin and NLR values and metastatic status) and nutritional variables (i.e., FAACT total score, VAS score for appetite loss and MNA^®^-based nutritional status) of cancer patients included in the prospective PreMiO study and their clinical outcomes (i.e., rehospitalization, onset of treatment-related toxicity, death, and disease progression) determined at the date of data cut-off of the retrospective NUTRIONCO study after patients’ follow-up.

Baseline clinical and nutritional data were categorized into binary variables as described in Section 2.

#### 3.2.1. Outcome: Disease Progression

A statistically significant association was found between baseline values of clinical and nutritional variables and disease progression in cancer patients at follow-up (*p* < 0.05), with the exception of the variable VAS for appetite loss (Table 4, *p* = 0.0911). In particular, a poor baseline nutritional status (MNA^®^ score ≤ 23.5) was present in 70.8% of patients with disease progression at follow-up, and a FAACT score < 30, indicative of anorexia, was detected in 70.3% of patients with disease progression at follow-up, vs. the 29.2% and 29.7% of patients with no disease progression at follow-up, respectively (Table 4). Similarly, baseline abnormal values of inflammation-related markers (albumin and NLR) were more common among patients with disease progression at follow-up (78.5% and 71.2%, respectively) (Table 4). Finally, 82.1% of cancer patients who presented disease progression at follow-up were diagnosed with a metastatic status at their first medical oncology visit (Table 4).

#### 3.2.2. Outcome: Patient’s Death

The outcome, “patient’s death”, was categorized in the database of the NUTRIONCO study as “death from disease progression” or as “death from other causes”. In the association analysis, the event was considered regardless of its cause. As shown in Table 5, a statistically significant association was found between values of all baseline clinical and nutritional variables measured at the first medical oncology visit and the patients’ death at follow-up (*p* < 0.0001). A baseline metastatic cancer and a poor nutritional status were particularly common (71% and 65.2%, respectively, Table 5) among patients who did not survive to the date of data cut-off of the NUTRIONCO database.

#### 3.2.3. Outcome: Patients’ Rehospitalization

A statistically significant association was found between a metastatic status diagnosed at the first medical oncology visit and patients’ rehospitalization at the date of data cut-off of the NUTRIONCO database (*p* < 0.0001, Table 6). In particular, only 34.9% of non-metastatic patients at baseline were found to have been rehospitalized at follow-up, vs. 52.7% of patients diagnosed with a metastatic cancer at their first medical oncology visit (Table 6). Baseline values of hemoglobin and NLR were also significantly associated with patients’ rehospitalization at the date of data cut-off of the NUTRIONCO database (*p* = 0.0376 and *p* = 0.0369, respectively) (Table 6).

#### 3.2.4. Outcome: Treatment-Related Toxicity

No statistically significant association was found between any of the baseline clinical and nutritional variables considered in the NUTRIONCO study and the treatment-related toxicity in cancer patients at the date of data cut-off of the NUTRIONCO database (Table 7).

### 3.3. Baseline Characteristics and Patients’ Survival: A Multivariate Analysis in the NUTRIONCO Study Population

**Figure 3:**
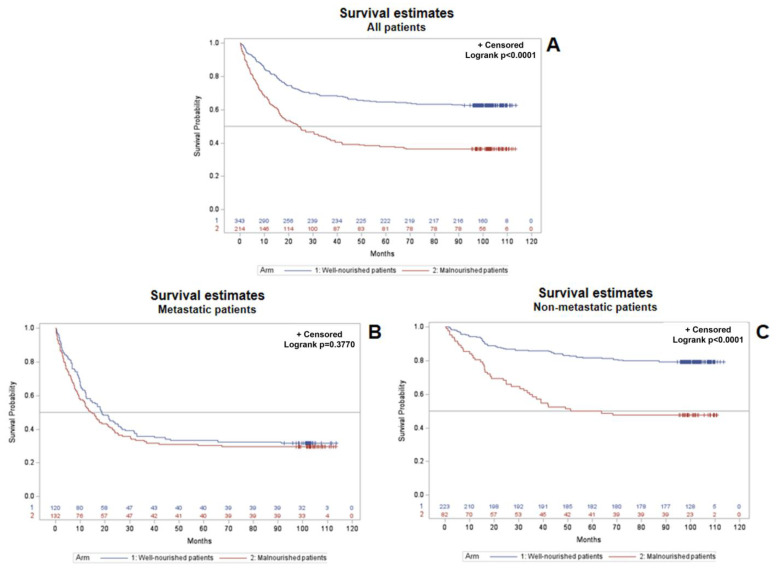
Kaplan–Meier curves with the log-rank test applied for the estimation of survival probability over time and comparison between curves, respectively. Overall survival probability estimated in the whole study population of cancer patients on the basis of their nutritional status (**A**), overall survival probability estimated in metastatic cancer patients on the basis of their nutritional status (**B**), overall survival probability estimated in non-metastatic cancer patients on the basis of their nutritional status (**C**).

Kaplan–Meier curves with the log-rank test were applied for the estimation of survival probability over time and the comparison between curves, respectively. Survival probability was lower in the overall population of malnourished patients vs. well-nourished patients (Log-rank, *p* < 0.0001) (Figure 3A). Patients’ stratification by metastatic status revealed that all metastatic patients had a substantially similar survival probability, regardless of their nutritional status (*p* = 0.6278) (Figure 3B). Conversely, the nutritional status significantly affected the survival probability of non-metastatic patients, with a lower survival probability in malnourished patients vs. well-nourished patients (*p* < 0.0001) (Figure 3C). Overall survival probability data highlight that the presence of malnutrition makes non-metastatic patients at risk of shorter survival, similarly to all metastatic patients, regardless of their nutritional status.

**Figure 4:**
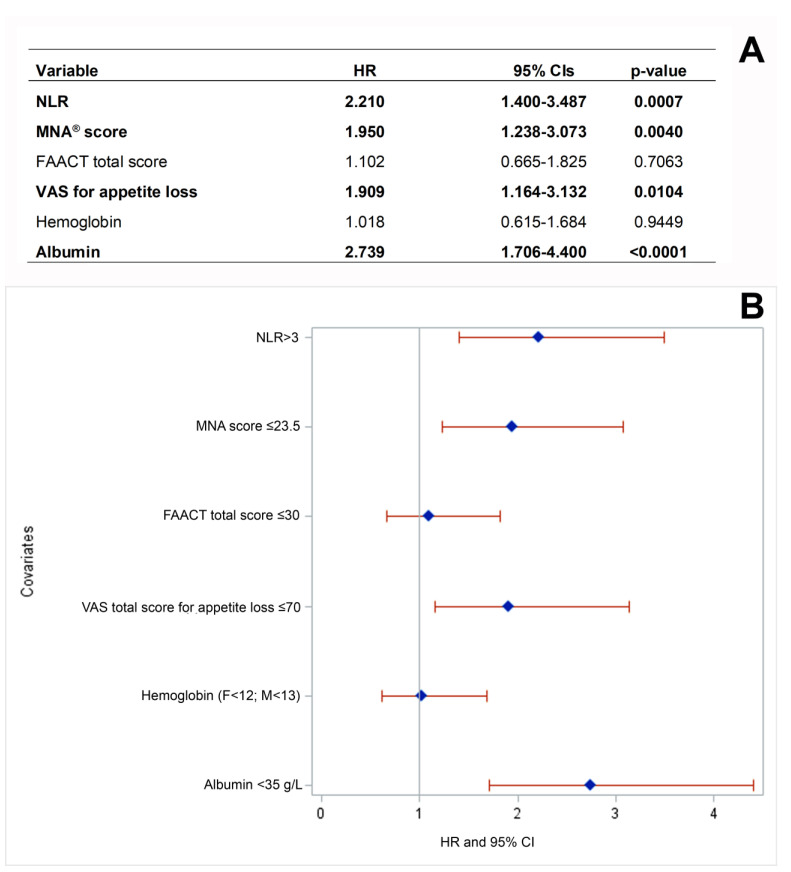
Multivariate analysis assessing the effect of independent variables on death of non-metastatic cancer patients (**A**) and forest plot of the independent variables (**B**). CI: Confidence interval; F: female; FAACT: Functional Assessment of Anorexia/Cachexia Treatment; HR: hazard ratio; M: male; MNA^®^: Mini Nutritional Assessment; NLR: neutrophil/lymphocyte ratio; VAS: visual analog scale. Cut-off values: FAACT score ≤ 30 for anorexia; VAS score ≤ 70 for appetite loss representative of anorexia; MNA^®^ scores: malnourished <17; at risk of malnutrition, 17 to 23.5; well-nourished >23.5; albumin < 35 g/L for hypoalbuminemia; hemoglobin < 12 g/dL in females and <13 g/dL in males for anemia; NLR > 3 for systemic inflammation. Variables independently associated with patients’ death are highlight in bold.

Considering the statistically significant difference in survival probability between well-nourished and malnourished non-metastatic patients described above, we further assessed the impact of baseline clinical and nutritional covariates on non-metastatic patients’ death at follow-up through a multivariate Cox regression analysis. Multivariate analysis confirmed that baseline malnutrition (*p* = 0.004) and VAS score for appetite loss (*p* = 0.0104), in addition to albumin < 35 g/L (*p* < 0.0001) and neutrophil/lymphocyte ratio >3 (*p* = 0.0007) were independently associated with death of non-metastatic patients at follow-up (Figure 4A,B).

## 4. Discussion

Poor nutritional status not only accelerates the progression of cancer, but also deeply impacts the tolerability and acceptability of anticancer treatments, creating a vicious cycle that involves overall QoL [13,23] and reduces the effectiveness of chemotherapy protocols and the final prognosis [13,24,25]. Nonetheless, malnutrition and cachexia in cancer patients are still undetected and underestimated in medical practice. Among others, a recent international quantitative survey based on 58 multiple-choice questions revealed that 23.7% of responders lacked confidence in their ability to provide care for patients with cancer cachexia, and only 29.1% of responders recognized a weight loss >5% from baseline as key criterion of cancer cachexia. Of note, only half of responders indicated that newly diagnosed patients with cancer should be screened for weight loss [26]. Another survey recently carried out on a sample of 300 Italian hospital medical oncologists revealed that almost all the respondents were aware of the nutritional–metabolic problems that a cancer patient could experience and of the importance of adequate nutrition during the therapeutic pathway [27]. However, the answers to the survey highlighted that nutritional support is not yet fully managed consistently with the available guidelines. Finally, a survey based on a 21-item questionnaire highlighted that digestive surgeons have a limited knowledge of basic aspects of clinical nutrition in gastrointestinal cancer patients, even if with some variability regarding the clinical practice in individual cases [28].

In this setting, the results of the NUTRIONCO study, indicating a significant association between the nutritional and inflammatory status of cancer patients at their first medical oncology visit and clinical outcomes at follow-up, further support the need for a greater awareness among oncologists of the importance of an early and effective assessment and management of malnutrition and cachexia in cancer patients.

Several patients with cancer cachexia suffer from systemic inflammation associated with high pro-inflammatory cytokine levels [29]. The sources of pro-inflammatory cytokines in cancer cachexia are numerous, and include tumor cells, tumor infiltrating cells along with peripheral tissue parenchymal cells, and associated infiltrating cells. This complex picture results in the establishment of a tumor–host interaction that promotes an imbalance in favor of the pro-inflammatory over the anti-inflammatory status [30]. The NLR and albumin variables we analyzed at basal level in the setting of the PreMiO and NUTRIONCO studies are considered markers of inflammation, which in turn leads to poor nutritional status, are routinely used in hospitals and are easily available and low-cost. They are known to play a role as prognostic and predictive factors in cancer patients [31,32]. In particular, high NLR values have been found to be associated with nutritional risk, independent of confounding variables, in hospitalized, unselected cancer patients [33]. NLR plays a key role in tumor initiation and progression, and may influence the response to anticancer treatments [34]. Among others, high NLR has been associated with an adverse OS in many solid tumors [35,36], and an elevated NLR has been negatively associated with OS and progression-free survival (PFS) in renal cell carcinoma (RCC) [37]. It is of major interest that pre-operative NLR has recently been demonstrated to be an inexpensive and easily accessible prognostic biomarker for non-metastatic RCC [38]. Consistently, our data show that high-baseline NLR (>3) was more common among cancer patients who did not survive at the cut-off date of the NUTRIONCO database (64.5%, Table 5), with a statistically significant association with the outcome “patient’s death” (*p* < 0.0001). High-baseline NLR was also more common among patients with disease progression at follow-up (71.2%, Table 4), and showed a statistically significant association with this outcome (*p* < 0.0001). The results of multivariate analysis further confirmed that high-baseline values of NLR are independently associated with patient death at follow-up (Figure 4).

Albumin is expressed as an acute-phase protein showing a negative correlation with the severity of the inflammatory response, and higher values of this protein predict longer OS in a cohort of cancer patients attending a cachexia support service [39]. A systematic review and meta-analysis including mainly retrospective studies investigated the prognostic value of markers of the systemic inflammatory response in patients with advanced cancer, revealing that albumin and NLR have an independent prognostic value across different tumor types [40]. Of note, this systematic review also highlighted a considerable variation among studies in the thresholds of albumin reported to have prognostic value. The data summarized above are consistent with our findings, showing that low-baseline albumin was more common among cancer patients who did not survive at the cut-off date of the NUTRIONCO database (72.4%, Table 5), with a statistically significant association with the outcome, “patient’s death” (*p* < 0.0001). Low-baseline albumin (<35 g/L) was also more common among patients with disease progression at follow-up (78.5%, Table 4), showing a statistically significant association with this outcome (*p* < 0.0001). The results of multivariate analysis further confirmed that low-baseline values of albumin are independently associated with patients’ death at follow-up (Figure 4), in line with the findings of other studies [39,40].

Anemia is common in cancer patients, and it is likely a multifactorial condition, correlating with poor performance status. Blood loss can be related to disease, particularly in patients affected by genitourinary, gastrointestinal, and gynecological cancers. Moreover, anticancer treatments can inhibit erythropoiesis, while causes of anemia have not yet been identified in a significant portion of cancer patients [41]. A multicenter retrospective cohort study recently demonstrated that low hemoglobin was positively correlated with all-cause mortality in patients with cancer cachexia, and this association was consistent across cancer subtypes [42]. These results are consistent with our data, showing a statistically significant association between baseline hemoglobin and patient death at cut-off date of the NUTRIONCO study database (Table 5).

Anorexia, defined as the loss of appetite or interest in food, is a frequently reported symptom in cancer patients and has been described as a robust negative predictor of long-term survival [43,44]. Consistently, the results of the NUTRIONCO study revealed a statistically significant association between both the baseline FACCT total score and the VAS score for appetite loss and patient death at follow-up (Table 5), as well as a significant association between the baseline FAACT total score and disease progression at follow-up (Table 4). Consistently with OS data (Figure 3) and with literature data [43,44], multivariate analysis further confirmed the impact of the independent variable VAS score for appetite loss (VAS score ≤ 70) on the death of non-metastatic patients (Figure 4).

With regard to malnutrition, a systematic review including 56 longitudinal studies concluded that baseline nutritional status assessed through the MNA^®^ is a potential prognostic factor for health and treatment outcomes at a later time point in patients of any age with any type of cancer and anticancer therapy [45]. In particular, malnutrition/risk of malnutrition significantly predicted a higher chance of mortality/poor OS, shorter PFS or time to progression, treatment maintenance, and QoL. Conversely, treatment toxicities and functional decline were not significantly predicted by MNA^®^ in adjusted analyses [45]. These data are consistent with our findings, revealing a statistically significant association between baseline malnutrition/risk of malnutrition assessed through the MNA^®^ and disease progression (Table 4, *p* < 0.0001) and patient death (Table 5, *p* < 0.0001) at follow-up, but not treatment toxicity (Table 7, *p* = 0.1654) and rehospitalization (Table 6, *p* = 0.2400). It is of major interest that the literature data mentioned above are also consistent with the results of the OS analysis in patients included in the NUTRIONCO study, indicating that malnutrition plays a major role in reducing the survival probability in overall study population (Figure 3A). A patient’s stratification on the basis of metastatic status further revealed that malnutrition plays a major role in reducing survival probability in non-metastatic patients (Figure 3C), while the survival of metastatic patients is not significantly affected by nutritional status (Figure 3B). Multivariate analysis further confirmed the impact of the independent variable malnutrition (MNA score ≤ 23.5) on the death of non-metastatic patients (Figure 4). These findings support the importance of the proactive, early management of malnutrition and cachexia in cancer patients, and in particular, in non-metastatic patients, from the perspective of a substantial improvement in their clinical outcomes.

Altogether, the results of the PreMiO [6] and NUTRIONCO studies highlight the importance of a timely and careful evaluation of nutritional risk in cancer patients, in particular those with non-metastatic disease, through early screenings and continuous application of supportive treatments. Strategies to limit or prevent cancer cachexia and improve nutritional status include support for cancer patients at nutritional risk with oral nutritional supplements, specific nutrients, and enteral or parenteral nutrition [46], i.e., relatively low-cost interventions that have not only clinical, but also financial benefits, with proven cost-effectiveness [47]. Other early strategies are based on the clinical management of symptoms that impede proper food intake (e.g., pain, depression, nausea, vomiting). In addition, several investigations are currently focused on appetite stimulation as a strategy to counteract anorexia in cancer [48,49]. Screening is the first step in the early detection and effective treatment of cancer cachexia. Incorporation of nutritional status evaluation and monitoring should therefore be regarded as a hallmark of good clinical practice in cancer treatment [50]. In the setting of early diagnosis and prevention of malnutrition in cancer patients, a parallel-pathway approach has been proposed, where medical oncology and clinical nutrition work in tandem from the onset of the natural history of the disease and during its progression. This parallel-pathway approach can offer patients a chance to prevent or delay the onset of cancer cachexia [10] through a series of individualized interventions. Notably, the PreMiO study was based on the direct involvement of adequately trained oncologists in the assessment of the nutritional status of cancer patients at their first medical oncology visit, including the subset of patients enrolled in the NUTRIONCO study, through the review of recent weight changes, appetite, inflammation, and through the application of validated scoring methods and criteria to detect malnutrition, anorexia, and cachexia [6,20,43]. The study results indicate that the active involvement of oncologists in nutritional assessment is possible and helpful in the early identification of malnutrition or of its risk, with potentially relevant clinical benefits, as also demonstrated elsewhere [51,52]. An expert panel has recently presented the Protocol for Nutritional Risk in Oncology (PRONTO), i.e., a standardized, simple and rapid approach for the identification and monitoring of nutritional risk in patients commencing and undergoing anticancer therapies [53]. The application of this novel tool is also feasible within the setting of a demanding oncology practice and when referral to dedicated nutritional services is unavailable.

The NUTRIONCO study was conducted at ten sites located in Italy, thus including a homogeneous cohort of patients in terms of genetic background, potentially affecting the nutritional phenotype. However, the group of patients was heterogeneous with respect to cancer type, thus increasing the generalizability of the findings of the NUTRIONCO study. These are some of the strengths of the study; however, it has also some limitations: firstly, the retrospective design of the study prevented us from inferring that the baseline clinical and nutritional variables increased the disease’s progression, rehospitalization, treatment toxicity, or mortality in patients with cancer. However, the study enabled us to find a significant association between several of the variables we selected at baseline and the outcomes we considered relevant in this cohort of patients. On this encouraging basis, prospective, longitudinal studies with an adequate number of participants and a low risk of potential bias are warranted in order to establish a causal relationship between baseline variables and final outcomes. Baseline nutritional status, anorexia and appetite loss data used for association analysis with clinical outcomes were evaluated through MNA^®^, FAACT and VAS, respectively, while baseline cachexia was evaluated in the PreMiO study based on Fearon criteria [6,15]. However, these nutritional screening tools cannot assess muscle mass or body composition, as is done with computed tomography (CT). Actually, CT-based body composition is a well-established prognostic marker in cancer patients [54]; nonetheless, the nutritional screening tools we used in the NUTRIONCO study are rapid, cost-effective, and widely accepted and recommended for the assessment of anorexia in the diagnosis of cancer anorexia-cachexia syndrome [20,43].

## 5. Conclusions

Malnutrition and risk of malnutrition are still under-recognized and underestimated issues in cancer patients, despite increasing evidence suggesting that their routine, early assessment and management would improve both short- and long-term clinical outcomes. In this respect, the NUTRIONCO and PreMiO consecutive studies, which shared a homogeneous subgroup of cancer patients, have shown that abnormal baseline clinical and nutritional variables are prevalent and statistically associated with death, rehospitalization, treatment toxicity, and disease progression at follow-up. Notably, a higher OS probability was found in the well-nourished general study population vs. malnourished patients. It is of major interest that patient stratification revealed that malnutrition decreased the survival probability in non-metastatic patients, but not in metastatic patients. Multivariate analysis confirmed that baseline malnutrition and VAS scores for appetite loss, in addition to albumin <35 g/L and NLR >3, were independently associated with the deaths of non-metastatic patients at follow-up. The results of this study will hopefully increase oncologists’ awareness of the unmet needs of cancer patients and of the importance of the early, proactive management of malnutrition and cachexia, with regard to a substantial improvement in clinical outcomes and QoL. Further efforts and changes in protocols for cancer patients’ management should be implemented with respect to this aim.

## Figures and Tables

**Table 1:** Baseline characteristics: demographics, nutritional and clinical features of patients included in the PreMiO and NUTRIONCO studies.

Parameter	Statistics	NUTRIONCO Study *n* = 571	PreMiO Study *n* = 1952
Age (years)	Mean (SD)	63.7 ± 12.7	62.7 ± 12.9
Gender	Male	262 (45.9%)	931 (47.7%)
	Female	309 (54.1%)	1021 (52.3%)
Weight (kg)	Mean (SD)	68.3 ± 13.8	68.4 ± 13.2
BMI (kg/m^2^)	Mean (SD)	24.8 ± 4.6	24.8 ± 4.4

BMI: Body Mass Index.

**Table 2:** Baseline characteristics: laboratory data and nutritional scores of patients included in the PreMiO and NUTRIONCO studies.

Parameter	Statistics	NUTRIONCO Study *n* = 571	PreMiO Study *n* = 1952
Albumin (g/L)	Mean (SD)	36.4 ± 6.0	36.3 ± 6.0
Hemoglobin (g/dL)	Mean (SD)	M: 12.7 ± 1.9	M: 12.4 ± 1.9
		F: 12.5 ± 1.6	F: 12.2 ± 2.0
NLR	Mean (SD)	3.7 ± 3.7	3.9 ± 4.7
FAACT total score	Mean (SD)	31.0 ± 5.6	29.9 ± 5.9
VAS anorexia	Mean (SD)	69.8 ± 21.1	67.0 ± 22.6

F: female; FAACT: Functional Assessment of Anorexia/Cachexia Treatment; M: male; NLR: neutrophil/lymphocyte ratio; VAS: visual analog scale.

**Table 3:** Number and percentage of patients included in the NUTRIONCO study above and below the cut-off values for FAACT total score and VAS for appetite loss by tumor type.

Tumor Type	FAACT ≤30	FAACT >30	VAS ≤70	VAS >70
Breast	45 (17.2%)	106 (34.2%)	57 (20.1%)	94 (32.8%)
Colorectal	41 (15.7%)	48 (15.5%)	46 (16.2%)	43 (15.0%)
Gastroesophageal	26 (10.0%)	6 (1.9%)	27 (9.5%)	5 (1.7%)
Genitourinary	31 (11.9%)	55 (17.7%)	33 (11.6%)	53 (18.5%)
Head and Neck	8 (3.1%)	15 (4.8%)	10 (3.5%)	13 (4.5%)
Hematologic	1 (0.4%)	1 (0.3%)	1 (0.4%)	1 (0.4%)
Liver and intrahepatic bile ducts	6 (2.3%)	4 (1.3%)	5 (1.8%)	5 (1.7%)
Other cancers	12 (4.6%)	11 (3.6%)	12 (4.2%)	11 (3.8%)
Other GI	8 (3.1%)	10 (3.2%)	15 (5.3%)	3 (1.1%)
Pancreas	30 (11.5%)	3 (1.0%)	24 (8.5%)	9 (3.1%)
Respiratory	50 (19.2%)	50 (16.1%)	51 (18.0%)	49 (17.1%)
Unknown primary	3 (1.2%)	1 (0.3%)	3 (1.1%)	1 (0.4%)

**Table 4:** Association analysis between baseline clinical and nutritional variables (PREMiO study), and the outcome, “disease progression”, at follow-up (NUTRIONCO study).

Parameter	Cut-Off Value/Status	Progression	Non-Progression	*p*-Value
Albumin (g/L)	<35	95 (78.5%)	26 (21.5%)	**<0.0001**
	≥35	107 (48.4%)	114 (51.6%)	
Hemoglobin (g/dL)	F < 12; M < 13	122 (69.3%)	54 (30.7%)	**0.0003**
	F ≥ 12; M ≥ 13	102 (47.0%)	115 (53.0%)	
NLR	>3	111 (71.2%)	45 (28.8%)	**<0.0001**
	1–3	111 (48.3%)	119 (51.7%)	
FAACT total score *	≤30	97 (70.3%)	41 (29.7%)	**<0.0001**
	>30	127 (49.4%)	130 (50.6%)	
VAS for appetite loss *	≤70	124 (60.8%)	80 (39.2%)	0.0911
	>70	100 (52.4%)	91 (47.6%)	
MNA^®^ *	Malnourished/At risk of malnutrition	109 (70.8%)	45 (29.2%)	**<0.0001**
	Well-nourished	114 (47.5%)	126 (52.5%)	
Mestastatic status	Metastatic	138 (82.1%)	30 (17.9%)	**<0.0001**
	Non-metastatic	86 (37.9%)	141 (62.1%)	

F: female; FAACT: Functional Assessment of Anorexia/Cachexia Treatment; M: Male; MNA^®^: Mini Nutritional Assessment; NLR: neutrophil/lymphocyte ratio; VAS: visual analog scale. * Cut-off values: FAACT score ≤ 30 for anorexia; VAS score ≤ 70 for appetite loss representative of anorexia; MNA^®^ scores: malnourished <17; at risk of malnutrition, 17 to 23.5; well-nourished >23.5; Albumin < 35 g/L for hypoalbuminemia; hemoglobin < 12 g/dL in females and <13 g/dL in males for anemia; NLR > 3 for systemic inflammation. *p*-values < 0.05 are highlighted in bold.

**Table 5:** Association analysis between baseline clinical and nutritional variables (PREMiO study) and the outcome, “patient’s death”, at follow-up (NUTRIONCO study).

Parameter	Cut-Off Value/Status	Dead	Alive	*p*-Value
Albumin (g/L)	<35	139 (72.4%)	53 (26.6%)	**<0.0001**
	≥35	120 (39.5%)	184 (60.5%)	
Hemoglobin (g/dL)	F < 12; M < 13	162 (62.1%)	99 (37.9%)	**<0.0001**
	F ≥ 12; M ≥ 13	116 (37.9%)	190 (62.1%)	
NLR	>3	156 (64.5%)	86 (35.5%)	**<0.0001**
	1–3	119 (37.5%)	198 (62.5%)	
FAACT total score *	≤30	138 (65.7%)	72 (34.3%)	**<0.0001**
	>30	140 (39.0%)	219 (61.0%)	
VAS for appetite loss *	≤70	167 (59.0%)	116 (41.0%)	**<0.0001**
	>70	111 (38.8%)	175 (61.2%)	
MNA^®^ *	Malnourished/At risk of malnutrition	146 (65.2%)	78 (34.8%)	**<0.0001**
	Well-nourished	131 (38.1%)	213 (61.9%)	
Mestastatic status	Metastatic	186 (71.0%)	76 (29.0%)	**<0.0001**
	Non-metastatic	92 (30.0%)	215 (70.0%)	

F: female; FAACT: Functional Assessment of Anorexia/Cachexia Treatment; M: Male; MNA^®^: Mini Nutritional Assessment; NLR: neutrophil/lymphocyte ratio; VAS: visual analog scale.* Cut-off values: FAACT score ≤ 30 for anorexia; VAS score ≤ 70 for appetite loss representative of anorexia; MNA^®^ scores: malnourished <17; at risk of malnutrition, 17 to 23.5; well-nourished >23.5; albumin < 35 g/L for hypoalbuminemia; Hemoglobin <12 g/dL in females and <13 g/dL in males for anemia; NLR > 3 for systemic inflammation. *p*-values < 0.05 are highlighted in bold.

**Table 6:** Association analysis between baseline clinical and nutritional variables (PREMiO study) and the outcome, “patient’s rehospitalization”, at follow-up (NUTRIONCO study).

Parameter	Cut-Off Value/Status	Rehospitalization	Non-Rehospitalization	*p*-Value
Albumin (g/L)	<35	93 (48.4%)	99 (51.6%)	0.1670
	≥35	128 (42.1%)	176 (57.9%)	
Hemoglobin (g/dL)	F < 12; M < 13	125 (47.9%)	136 (52.1%)	**0.0376**
	F ≥ 12; M ≥ 13	120 (39.2%)	186 (60.8%)	
NLR	>3	116 (47.9%)	126 (52.1%)	**0.0369**
	1–3	124 (39.1%)	193 (60.9%)	
FAACT total score *	≤30	99 (47.1%)	111 (52.9%)	0.1323
	>30	146 (40.7%)	213 (59.3%)	
VAS for appetite loss *	≤70	125 (44.2%)	158 (55.8%)	0.5942
	>70	120 (42.0%)	166 (58.0%)	
MNA^®^ *	Malnourished/at risk of malnutrition	103 (46.0%)	121 (54.0%)	0.2400
	Well-nourished	141 (41.0%)	203 (59.0%)	
Mestastatic status	Metastatic	138 (52.7%)	124 (47.3%)	**<0.0001**
	Non-metastatic	107 (34.9%)	200 (65.1%)	

F: female; FAACT: Functional Assessment of Anorexia/Cachexia Treatment; M: Male; MNA^®^: Mini Nutritional Assessment; NLR: neutrophil/lymphocyte ratio; VAS: visual Analog Scale. * Cut-off values: FAACT score ≤ 30 for anorexia; VAS score ≤ 70 for appetite loss representative of anorexia; MNA^®^ scores: malnourished < 17; at risk of malnutrition, 17 to 23.5; well-nourished > 23.5; albumin < 35 g/L for hypoalbuminemia; hemoglobin < 12 g/dL in females and <13 g/dL in males for anemia; NLR > 3 for systemic inflammation. *p*-values < 0.05 are highlighted in bold.

**Table 7:** Association analysis between baseline clinical and nutritional variables (PREMiO study) and the outcome, “treatment toxicity”, at follow-up (NUTRIONCO study).

Parameter	Cut-Off Value/Status	Toxicity	Non-Toxicity	*p*-Value
Albumin (g/L)	<35	65 (33.9%)	127 (66.2%)	0.7035
	≥35	108 (35.5%)	196 (64.5%)	
Hemoglobin (g/dL)	F < 12; M < 13	82 (31.4%)	179 (68.6%)	0.1244
	F ≥ 12; M ≥ 13	115 (37.6%)	191 (62.4%)	
NLR	>3	85 (35.1%)	157 (64.9%)	0.7950
	1–3	108 (34.1%)	209 (65.9%)	
FAACT total score *	≤30	67 (31.9%)	143 (68.1%)	0.2974
	>30	130 (36.2%)	229 (63.8%)	
VAS for appetite loss *	≤70	97 (34.3%)	186 (65.7%)	0.8628
	>70	100 (35.0%)	186 (65.0%)	
MNA^®^ *	Malnourished/at risk of malnutrition	70 (31.2%)	154 (68.8%)	0.1654
	Well-nourished	127 (36.9%)	217 (63.1%)	
Mestastatic status	Metastatic	93 (35.5%)	169 (64.5%)	0.6856
	Non-metastatic	104 (33.9%)	203 (66.1%)	

F: female; FAACT: Functional Assessment of Anorexia/Cachexia Treatment; M: Male; MNA^®^: Mini Nutritional Assessment; NLR: neutrophil/lymphocyte ratio; VAS: visual analog scale.* Cut-off values: FAACT score ≤ 30 for anorexia; VAS score ≤ 70 for appetite loss representative of anorexia; MNA^®^ scores: malnourished <17; at risk of malnutrition, 17 to 23.5; well-nourished >23.5; albumin < 35 g/L for hypoalbuminemia; Hemoglobin < 12 g/dL in females and <13 g/dL in males for anemia; NLR > 3 for systemic inflammation.

## Data Availability

The datasets used and analyzed during the present study are available from the corresponding author on reasonable request.

## References

[1] World Health Organization https://www.who.int/news-room/fact-sheets/detail/malnutrition.

[2] Zimta A.A., Tigu A.B., Muntean M., Cenariu D., Slaby O., Berindan-Neagoe I. (2019). Molecular Links between Central Obesity and Breast Cancer. Int. J. Mol. Sci..

[3] Saunders J., Smith T. (2010). Malnutrition: Causes and consequences. Clin. Med..

[4] Bossi P., Delrio P., Mascheroni A., Zanetti M. (2021). The Spectrum of Malnutrition/Cachexia/Sarcopenia in Oncology According to Different Cancer Types and Settings: A Narrative Review. Nutrients.

[5] Cederholm T., Barazzoni R., Austin P., Ballmer P., Biolo G., Bischoff S.C., Compher C., Correia I., Higashiguchi T., Holst M. (2017). ESPEN guidelines on definitions and terminology of clinical nutrition. Clin. Nutr..

[6] Muscaritoli M., Lucia S., Farcomeni A., Lorusso V., Saracino V., Barone C., Plastino F., Gori S., Magarotto R., Carteni G. (2017). Prevalence of malnutrition in patients at first medical oncology visit: The PreMiO study. Oncotarget.

[7] Martin L., Muscaritoli M., Bourdel-Marchasson I., Kubrak C., Laird B., Gagnon B., Chasen M., Gioulbasanis I., Wallengren O., Voss A.C. (2021). Diagnostic criteria for cancer cachexia: Reduced food intake and inflammation predict weight loss and survival in an international, multi-cohort analysis. J. Cachexia Sarcopenia Muscle.

[8] Marceca G.P., Londhe P., Calore F. (2020). Management of Cancer Cachexia: Attempting to Develop New Pharmacological Agents for New Effective Therapeutic Options. Front. Oncol..

[9] van der Meij B.S., Teleni L., McCarthy A.L., Isenring E.A. (2020). Cancer Cachexia: An Overview of Diagnostic Criteria and Therapeutic Approaches for the Accredited Practicing Dietitian. J. Hum. Nutr. Diet..

[10] Muscaritoli M., Molfino A., Gioia G., Laviano A., Rossi Fanelli F. (2011). The “parallel pathway”: A novel nutritional and metabolic approach to cancer patients. Intern. Emerg. Med..

[11] Ni J., Zhang L. (2020). Cancer Cachexia: Definition, Staging, and Emerging Treatments. Cancer Manag. Res..

[12] O’Connell T.M., Golzarri-Arroyo L., Pin F., Barreto R., Dickinson S.L., Couch M.E., Bonetto A. (2021). Metabolic Biomarkers for the Early Detection of Cancer Cachexia. Front. Cell. Dev. Biol..

[13] Ryan A.M., Prado C.M., Sullivan E.S., Power D.G., Daly L.E. (2019). Effects of weight loss and sarcopenia on response to chemotherapy, quality of life, and survival. Nutrition.

[14] Ryan A.M., Power D.G., Daly L., Cushen S.J., Ní Bhuachalla Ē., Prado C.M. (2016). Cancer-associated malnutrition, cachexia and sarcopenia: The skeleton in the hospital closet 40 years later. Proc. Nutr. Soc..

[15] Fearon K., Strasser F., Anker S.D., Bosaeus I., Bruera E., Fainsinger R.L., Jatoi A., Loprinzi C., MacDonald N., Mantovani G. (2011). Definition and Classification of Cancer Cachexia: An International Consensus. Lancet Oncol..

[16] Meyer F., Valentini L. (2019). Disease-Related Malnutrition and Sarcopenia as Determinants of Clinical Outcome. Visc. Med..

[17] Molfino A., Emerenziani S., Tonini G., Santini D., Gigante A., Guarino M.P.L., Nuglio C., Imbimbo G., La Cesa A., Cicala M. (2023). Early impairment of food intake in patients newly diagnosed with cancer. Front. Nutr..

[18] Reber E., Schönenberger K.A., Vasiloglou M.F., Stanga Z. (2021). Nutritional Risk Screening in Cancer Patients: The First Step Toward Better Clinical Outcome. Front. Nutr..

[19] Muscaritoli M., Anker S.D., Argilés J., Aversa Z., Bauer J.M., Biolo G., Boirie Y., Bosaeus I., Cederholm T., Costelli P. (2010). Consensus definition of sarcopenia, cachexia and pre-cachexia: Joint document elaborated by Special Interest Groups (SIG) “cachexia-anorexia in chronic wasting diseases” and “nutrition in geriatrics”. Clin. Nutr..

[20] Blauwhoff-Buskermolen S., Ruijgrok C., Ostelo R.W., de Vet H.C.W., Verheul H.M.W., de van der Schueren M.A.E., Langius J.A.E. (2016). The assessment of anorexia in patients with cancer: Cut-off values for the FAACT-A/CS and the VAS for appetite. Support. Care Cancer.

[21] Grenader T., Waddell T., Peckitt C., Oates J., Starling N., Cunningham D., Bridgewater J. (2016). Prognostic value of neutrophil-to-lymphocyte ratio in advanced oesophago-gastric cancer: Exploratory analysis of the REAL-2 trial. Ann. Oncol..

[22] McGovern J., Dolan R.D., Skipworth R.J., Laird B.J., McMillan D.C. (2022). Cancer cachexia: A nutritional or a systemic inflammatory syndrome?. Br. J. Cancer.

[23] Gellrich N.C., Handschel J., Holtmann H., Krüskemper G. (2015). Oral cancer malnutrition impacts weight and quality of life. Nutrients.

[24] Bolte F.J., McTavish S., Wakefield N., Shantzer L., Hubbard C., Krishnaraj A., Novicoff W., Gentzler R.D., Hall R.D. (2022). Association of sarcopenia with survival in advanced NSCLC patients receiving concurrent immunotherapy and chemotherapy. Front. Oncol..

[25] Chen L., Qi Y., Kong X., Su Z., Wang Z., Wang X., Du Y., Fang Y., Li X., Wang J. (2022). Nutritional Risk Index Predicts Survival in Patients with Breast Cancer Treated with Neoadjuvant Chemotherapy. Front. Nutr..

[26] Baracos V.E., Coats A.J., Anker S.D., Sherman L., Klompenhouwer T., International Advisory Board, and Regional Advisory Boards for North America, Europe, and Japan (2022). Identification and management of cancer cachexia in patients: Assessment of healthcare providers’ knowledge and practice gaps. J. Cachexia Sarcopenia Muscle.

[27] Muscaritoli M., Corsaro E., Molfino A. (2021). Awareness of Cancer-Related Malnutrition and Its Management: Analysis of the Results from a Survey Conducted Among Medical Oncologists. Front. Oncol..

[28] Durán-Poveda M., Suárez-de-la-Rica A., Cancer Minchot E., Ocón-Bretón J., Sánchez-Pernaute A., Rodríguez-Caravaca G. (2022). Knowledge and Practices of Digestive Surgeons concerning Specialized Nutritional Support in Cancer Patients: A Survey Study. Nutrients.

[29] Clamon G., Byrne M.M., Talbert E.E. (2022). Inflammation as a Therapeutic Target in Cancer Cachexia. Cancers.

[30] de Matos-Neto E.M., Lima J.D., de Pereira W.O., Figuerêdo R.G., Riccardi D.M., Radloff K., das Neves R.X., Camargo R.G., Maximiano L.F., Tokeshi F. (2015). Systemic Inflammation in Cachexia-Is Tumor Cytokine Expression Profile the Culprit?. Front. Immunol..

[31] Kaya T., Açıkgöz S.B., Yıldırım M., Nalbant A., Altaş A.E., Cinemre H. (2019). Association between neutrophil-to-lymphocyte ratio and nutritional status in geriatric patients. J. Clin. Lab. Anal..

[32] Hsueh W.H., Hsueh S.W., Yeh K.Y., Hung Y.S., Ho M.M., Lin S.Y., Tseng C.K., Hung C.Y., Chou W.C. (2022). Albumin and Neutrophil-to-Lymphocyte Ratio Score in Neoadjuvant Concurrent Chemoradiotherapy for Esophageal Cancer: Comparison with Prognostic Nutritional Index. In Vivo.

[33] Siqueira J.M., Soares J.D.P., Borges T.C., Gomes T.L.N., Pimentel G.D. (2021). High neutrophil to lymphocytes ratio is associated with nutritional risk in hospitalised, unselected cancer patients: A cross-sectional study. Sci. Rep..

[34] Guthrie G.J., Charles K.A., Roxburgh C.S., Horgan P.G., McMillan D.C., Clarke S.J. (2013). The systemic inflammation-based neutrophil-lymphocyte ratio: Experience in patients with cancer. Crit. Rev. Oncol. Hematol..

[35] Templeton A.J., McNamara M.G., Šeruga B., Vera-Badillo F.E., Aneja P., Ocaña A., Leibowitz-Amit R., Sonpavde G., Knox J.J., Tran B. (2014). Prognostic role of neutrophil-to-lymphocyte ratio in solid tumors: A systematic review and meta-analysis. J. Natl. Cancer Inst..

[36] Corbeau I., Jacot W., Guiu S. (2020). Neutrophil to Lymphocyte Ratio as Prognostic and Predictive Factor in Breast Cancer Patients: A Systematic Review. Cancers.

[37] Nunno V.D., Mollica V., Gatto L., Santoni M., Cosmai L., Porta C., Massari F. (2019). Prognostic impact of neutrophil-to-lymphocyte ratio in renal cell carcinoma: A systematic review and meta-analysis. Immunotherapy.

[38] Allenet C., Klein C., Rouget B., Margue G., Capon G., Alezra E., Blanc P., Estrade V., Bladou F., Robert G. (2022). Can Pre-Operative Neutrophil-to-Lymphocyte Ratio (NLR) Help Predict Non-Metastatic Renal Carcinoma Recurrence after Nephrectomy? (UroCCR-61 Study). Cancers.

[39] Bland K.A., Zopf E.M., Harrison M., Ely M., Cormie P., Liu E., Dowd A., Martin P. (2021). Prognostic Markers of Overall Survival in Cancer Patients Attending a Cachexia Support Service: An Evaluation of Clinically Assessed Physical Function, Malnutrition and Inflammatory Status. Nutr. Cancer.

[40] Dolan R.D., McSorley S.T., Horgan P.G., Laird B., McMillan D.C. (2017). The role of the systemic inflammatory response in predicting outcomes in patients with advanced inoperable cancer: Systematic review and meta-analysis. Crit. Rev. Oncol. Hematol..

[41] Ludwig H., Van Belle S., Barrett-Lee P., Birgegård G., Bokemeyer C., Gascón P., Kosmidis P., Krzakowski M., Nortier J., Olmi P. (2004). The European Cancer Anaemia Survey (ECAS): A large, multinational, prospective survey defining the prevalence, incidence, and treatment of anaemia in cancer patients. Eur. J. Cancer.

[42] Zhang X.W., Zhang Q., Song M.M., Zhang K.P., Zhang X., Ruan G.T., Yang M., Ge Y.Z., Tang M., Li X.R. (2022). The prognostic effect of hemoglobin on patients with cancer cachexia: A multicenter retrospective cohort study. Support. Care Cancer.

[43] Molfino A., de van der Schueren M.A.E., Sánchez-Lara K., Milke P., Amabile M.I., Imbimbo G., Di Lazzaro L., Cavuto S., Ronzani G., Snegovoy A. (2021). Cancer-associated anorexia: Validity and performance overtime of different appetite tools among patients at their first cancer diagnosis. Clin. Nutr..

[44] Martin L., Watanabe S., Fainsinger R., Lau F., Ghosh S., Quan H., Atkins M., Fassbender K., Downing G.M., Baracos V. (2010). Prognostic factors in patients with advanced cancer: Use of the patient-generated subjective global assessment in survival prediction. J. Clin. Oncol..

[45] Torbahn G., Strauss T., Sieber C.C., Kiesswetter E., Volkert D. (2020). Nutritional status according to the mini nutritional assessment (MNA)^®^ as potential prognostic factor for health and treatment outcomes in patients with cancer-a systematic review. BMC Cancer.

[46] de van der Schueren M.A.E., Laviano A., Blanchard H., Jourdan M., Arends J., Baracos V.E. (2018). Systematic review and meta-analysis of the evidence for oral nutritional intervention on nutritional and clinical outcomes during chemo(radio)therapy: Current evidence and guidance for design of future trials. Ann. Oncol..

[47] Schuetz P., Sulo S., Walzer S., Krenberger S., Brunton C. (2022). Nutritional support during the hospital stay is cost-effective for preventing adverse outcomes in patients with cancer. Front. Oncol..

[48] Nishie K., Sato S., Hanaoka M. (2022). Anamorelin for cancer cachexia. Drugs Today.

[49] Lim Y.L., Teoh S.E., Yaow C.Y.L., Lin D.J., Masuda Y., Han M.X., Yeo W.S., Ng Q.X. (2022). A Systematic Review and Meta-Analysis of the Clinical Use of Megestrol Acetate for Cancer-Related Anorexia/Cachexia. J. Clin. Med..

[50] Muscaritoli M., Arends J., Aapro M. (2019). From guidelines to clinical practice: A roadmap for oncologists for nutrition therapy for cancer patients. Ther. Adv. Med. Oncol..

[51] Ravasco P., Monteiro-Grillo I., Marques Vidal P., Camilo M.E. (2005). Impact of nutrition on outcome: A prospective randomized controlled trial in patients with head and neck cancer undergoing radiotherapy. Head Neck.

[52] Isenring E.A., Capra S., Bauer J.D. (2004). Nutrition intervention is beneficial in oncology outpatients receiving radiotherapy to the gastrointestinal or head and neck area. Br. J. Cancer.

[53] Muscaritoli M., Bar-Sela G., Battisti N.M.L., Belev B., Contreras-Martínez J., Cortesi E., de Brito-Ashurst I., Prado C.M., Ravasco P., Yalcin S. (2023). Oncology-Led Early Identification of Nutritional Risk: A Pragmatic, Evidence-Based Protocol (PRONTO). Cancers.

[54] Bates D.D.B., Pickhardt P.J. (2022). CT-Derived Body Composition Assessment as a Prognostic Tool in Oncologic Patients: From Opportunistic Research to Artificial Intelligence-Based Clinical Implementation. AJR Am. J. Roentgenol..

